# Biochemical and Molecular Markers Among Prediabetic and Type 2 Diabetes Mellitus Patients

**DOI:** 10.5812/ijem-171089

**Published:** 2026-06-13

**Authors:** Danya Omed Ghareeb, Heshu Sulaiman Rahman

**Affiliations:** 1 Department of Medical Laboratory Sciences, College of Science, Charmo University, Chamchamal, Sulaimaniyah, Iraq; 2 Branch of Basic Medical Sciences, College of Medicine, University of Sulaimani, Sulaimaniyah, Iraq

**Keywords:** Diabetes, Prediabetes, Glycemic Control, Molecular Markers, Biochemical Tests

## Abstract

**Background:**

Type 2 diabetes mellitus (T2DM) progresses through prediabetes (PD), which is characterized by insulin resistance and β-cell dysfunction. More than 50% of T2DM cases remain undiagnosed, underscoring the need for specific molecular markers beyond standard diagnostic tests.

**Objectives:**

This study aimed to compare sociodemographic, biochemical, and molecular profiles among groups to improve risk stratification.

**Methods:**

This cross-sectional study recruited 90 participants, including healthy controls (HC), individuals with PD, and patients with T2DM (n = 30 each), at Smart Health Tower, Sulaymaniyah, Iraq, from January to June 2025. Approximately 6.0 mL of blood was collected from each participant and analyzed for glycated hemoglobin (HbA1C), random blood glucose (RBG), C-peptide, lipid profile, urea, and creatinine, as well as molecular markers, including micro-ribonucleic acids (miRNAs) such as miRNA-126 and miRNA-132. Variables were subsequently compared among the groups.

**Results:**

Glycemic markers differed markedly among groups, with HbA1C and RBG highest in T2DM (P < 0.050). In PD, C-peptide (3.04 ± 1.33 ng/mL, P < 0.010) and high-density lipoprotein (HDL) (48.2 ± 12.3 mg/dL, P = 0.028) were highest. Renal markers showed the lowest creatinine level in T2DM (0.72 ± 0.18 mg/dL, P < 0.010), whereas urea levels were comparable among groups (P > 0.05). In patients with T2DM, correlations were observed between RBG and HbA1C (r = 0.792, P < 0.001), C-peptide and triglycerides (r = 0.598, P < 0.001), and HbA1C and creatinine (r = -0.452, P = 0.012). In the PD group, RBG correlated with C-peptide (r = 0.387, P = 0.035). In contrast, miRNA-132 expression was lowest in HC (0.90 ± 0.63; 95% CI, 0.67 - 1.14), significantly increased in PD (2.50 ± 1.86; 95% CI, 1.74 - 3.26), and highest in T2DM (3.56 ± 2.04; 95% CI, 2.80 - 4.32), with highly significant differences between HC and PD and between HC and T2DM (P < 0.001), as well as between PD and T2DM (P = 0.045). Additionally, miRNA-126 expression was lowest in HC (1.22 ± 0.52; 95% CI, 1.03 - 1.42), moderately elevated in T2DM (1.29 ± 0.85; 95% CI, 0.97 - 1.61), and highest in PD (1.70 ± 0.74; 95% CI, 1.41 - 1.98), with significant differences between PD and HC (P = 0.021) and between PD and T2DM (P = 0.029), while no significant difference was observed between HC and T2DM (P = 0.252).

**Conclusions:**

The identified interconnections among glycemic, lipid, and renal indicators underscore the importance of early identification, comprehensive biochemical evaluation, and timely care during the PD phase to prevent progression to overt DM and its associated complications.

## 1. Background

Type 2 diabetes mellitus (T2DM) is characterized by persistently high blood glucose (BG), particularly after carbohydrate intake. Unlike type 1 diabetes mellitus (T1DM), which involves insulin deficiency, most patients with T2DM have elevated fasting or post-glucose insulin levels unless beta (β)-cell failure occurs. Insulin resistance (IR) explains the persistence of high glucose levels despite sufficient insulin (1). Globally, approximately 50% of people aged 20 - 79 years with diabetes mellitus (DM) are unaware of their condition; this varies by region, with approximately one-third undiagnosed in high-income countries and more than 75% undiagnosed in low-income countries (2). Prediabetes (PD) is an intermediate stage between normal glucose tolerance (normoglycemia) and T2DM (3), with a high risk of progression to T2DM (4). Prediabetes is identified using fasting blood glucose (FBG), glycated hemoglobin (HbA1C), or 2-hour post-load BG (2h-PBG) tests, and it indicates a high risk of developing DM (5). A better understanding of PD enables earlier identification and intervention, potentially reducing the development of DM. However, varying definitions and screening criteria across guidelines lead to wide differences in prevalence estimates (6). Type 2 diabetes mellitus, the most prevalent form of DM, accounts for approximately 90% of all DM cases worldwide. Although it primarily affects middle-aged and older adults, its incidence is increasing in younger populations because of rising rates of obesity, physical inactivity, and unhealthy dietary habits (7). Alarmingly, more than 50% of T2DM cases are estimated to be undiagnosed. Therefore, identifying reliable biochemical and molecular markers is essential for facilitating early diagnosis of both PD and T2DM. This would enable the development of noninvasive, sensitive, and cost-effective screening tools, ultimately supporting timely intervention and reducing the risk of complications (8).

Micro-ribonucleic acids (miRNAs) are short noncoding RNAs (21 - 23 nucleotides) that regulate gene expression post-transcriptionally. In DM, many miRNAs are dysregulated, making them potential markers for T2DM risk (9). Micro-ribonucleic acids regulate cell growth, differentiation, proliferation, and death and act as novel signaling molecules for intercellular communication. Some are improved in individuals with PD. When combined with HbA1C, plasma miRNA levels may help assess early T2DM risk and prevent progression (10). MicroRNA-126 is significantly downregulated in DM compared with PD, with the highest levels in nondiabetic individuals, suggesting strong potential as a diagnostic biomarker for PD and DM (11). Modulating the amount of miR-132 in the body could be used to treat DM. In vivo tests show that antagomir-132 administration lowers BG levels and increases insulin production. RNA-based treatments, such as antagomirs targeting miR-132, control glucose metabolism and enhance β-cell activity. These findings indicate that miR-132 could be a promising target for improving BG control in patients with diabetes (12). Traditional markers range from qualitative to quantitative and include random blood glucose (RBG), FBG, HbA1C, and the 2-hour oral glucose tolerance test (OGTT) as primary markers, and C-peptide, lipid profile, and renal function tests as secondary markers. However, these markers have limitations, including moderate sensitivity and specificity. Therefore, combining several markers may more precisely identify individuals at high risk of developing PD and subsequently progressing to DM (10, 13).

## 2. Objectives

This study aimed to compare sociodemographic characteristics, glycemic markers, lipid profiles, and renal function markers among the HC, PD, and T2DM groups. It also aimed to quantify the expression of circulating miRNA-126 and miRNA-132 and compare their profiles across glycemic stages.

## 3. Methods

### 3.1. Study Design and Setting

This prospective cross-sectional study was conducted among 90 individuals who visited Smart Health Tower, Sulaymaniyah, Iraq, for routine medical checkups, treatment, or regular follow-up. Data collection and experimental procedures were conducted sequentially from January to June 2025, in accordance with the Strengthening the Reporting of Observational Studies in Epidemiology (STROBE) guidelines.

### 3.2. Participant Selection and Sampling Technique

A total of 90 participants, including HC individuals, individuals with PD, and patients diagnosed with T2DM (n = 30 each), were enrolled using a consecutive sampling method. According to the American Diabetes Association criteria (14), HC individuals were defined by fasting plasma glucose (FPG) of < 100 mg/dL, 2hppBS of < 140 mg/dL, HbA1C of < 5.7%, and a postprandial glucose level of 70 - 90 mg/dL approximately 5 hours after a meal. Prediabetes was defined by FPG of 100 - 125 mg/dL, 2hppBS of 140 - 199 mg/dL, and HbA1C of 5.7% - 6.4%. Finally, T2DM was defined by RBG of ≥ 200 mg/dL, FBG of ≥ 126 mg/dL on 2 separate tests, OGTT of 200 mg/dL, and HbA1C of ≥ 6.5% (14).

### 3.3. Inclusion Criteria

Eligible participants were aged 30 - 60 years and were either HC individuals, individuals with PD, or patients with T2DM. Participants received clinical diagnoses from a competent physician, which were subsequently validated using conventional laboratory procedures.

### 3.4. Exclusion Criteria

Individuals with T1DM; a history of significant comorbidities, including hypertension, epilepsy, cancer, end-stage renal disease, liver failure, or autoimmune disorders; pregnant, breastfeeding, or postpartum women; and individuals receiving continuous pharmaceutical treatment for diseases not linked to DM control were excluded.

### 3.5. Questionnaire

A self-developed questionnaire, validated by 5 experts in the field to improve accuracy, accessibility, and reliability, was used to collect participants’ sociodemographic data (age, gender, marital status, occupation, and residency), clinical characteristics (family history of diabetes, disease duration, symptoms, chronic conditions, and diabetes therapies), BG monitoring frequency, and biochemical parameters (glycemic markers, lipid profile, and renal function tests).

### 3.6. Blood Sample Collection and Laboratory Analysis

Approximately 6.0 mL of venous blood was collected from each participant. Of this, 2 mL was placed into plain tubes and centrifuged at 3000 revolutions for 10 minutes to obtain plasma, which was aliquoted and stored at -20°C for molecular analysis. Another 3 mL of blood was placed in a serum separation tube to obtain serum, which was used to assess C-peptide, the lipid profile (triglyceride, total cholesterol, low-density lipoprotein, and high-density lipoprotein), and renal function tests (urea and creatinine) using a biochemical analyzer (Cobas e 402 and c 503, Switzerland). The remaining 1.0 mL of blood was used immediately to assess BG levels and HbA1C.

### 3.7. Molecular Study

Total RNA was extracted using the NZY miRNA Isolation & RNA Clean-up Kit (NZYtech, catalog No. MB45801, Germany), which enables purification of all types of RNA, including miRNA, while allowing simultaneous removal of protein and DNA contaminants. Briefly, frozen plasma samples were thawed gradually on ice to avoid thermal damage to RNA. Samples were then centrifuged at 10000 × g for 10 - 15 minutes at room temperature to remove residual platelets and cellular debris. Several modifications were made to optimize the RNA extraction protocol for plasma samples because the kit was primarily intended for cells and tissues. A starting volume of 200 μL of plasma was used, and extraction was performed according to the manufacturer’s protocol. To enhance RNA concentration, the elution volume was reduced to 25 μL. In addition, the eluate was reapplied to the silica membrane column, followed by an additional centrifugation step, to increase the concentration of recovered total RNA, including miRNAs. RNA concentration and purity were subsequently evaluated using a NanoDrop spectrophotometer (Thermo Fisher Scientific, USA). U6 small nuclear RNA (snRNA) served as the endogenous reference gene for normalization in reverse transcriptase-quantitative polymerase chain reaction (RT-qPCR) assays. U6 is a spliceosomal component known for stable expression under various physiological conditions and has been shown to provide reliable, consistent expression in miRNA biomarker studies (15). PCR amplification was performed using the one-step NZYSpeedy RT-qPCR Green Kit (NZYtech), which enables reverse transcription within a single tube. The 2× master mix contains SYBR Green dye, dNTPs, DNA polymerase, and optimized reaction buffer components in a total volume of 20 μL (Table 1), along with self-developed reverse and forward primers obtained from Bioneer Corporation, South Korea (Table 2). The PCR cycling conditions included reverse transcription at 45°C for 10 minutes, polymerase activation at 95°C for 5 minutes, denaturation at 95°C for 10 seconds, and annealing with extension at 60°C for 45 seconds. Finally, relative miRNA expression was quantified using the Pfaffl method, which provides an efficiency-corrected framework for comparing gene expression levels between test and reference samples.

**Table 1.** Reverse Transcription-Quantitative Polymerase Chain Reaction (RT-qPCR) Components

Material	Reaction (μL)
**One-step NZYSpeedy qPCR Green master mix (2×)**	10
**10 μM forward primer**	0.8
**10 μM reverse primer**	0.8
**NZYRT mix**	0.8
**Adapter**	0.8
**Sample**	1 - 5
**Deionized distilled water**	5
**Final volume**	20

**Table 2.** Primer Sequence Used for the Reverse Transcription-Quantitative Polymerase Chain Reaction Assay ^a^

Primer name	Primer sequence
**hsa-miR-126b-5p F**	5'TCTACCGTTCATTACTCTAA3'
**hsa-miR-126b-5p R**	5'GTCGTATCCAGTGCAGGGTCCGAGGTATTCGCACTGGATACGACTCATAAC3'
**hsa-miR-132 - 5p F**	5'TAA TGC TTG CCT ACG3'
**hsa-miR-132 - 5p R**	5'GTGCAGGGTCCGAGGT3'

^a^ Abbreviation: miR, Micro-ribonucleic acid.

### 3.8. Statistical Analysis

The Statistical Package for the Social Sciences (IBM, Chicago, USA, version 26) was used for data analysis. Data normality was assessed using the Kolmogorov-Smirnov test. Continuous variables were expressed as mean ± standard deviation (SD), and categorical variables were expressed as number and percentage. Analysis of variance (ANOVA) was used for comparisons between groups (HC, PD, and T2DM) when data were normally distributed (such as age), whereas categorical variables were compared using the chi-square test (such as gender, marital status, occupation, and residency). For nonparametric comparisons between groups, the Kruskal-Wallis test was applied (such as BMI), along with the Mann-Whitney U test (such as disease duration, HbA1C, RBG, C-peptide, lipid profile, renal biomarkers, and molecular biomarkers). Correlations between biochemical markers (HbA1C, RBG, C-peptide, TG, TC, HDL, LDL, urea, and creatinine) and molecular markers (miRNA-126 and miRNA-132) were evaluated using Spearman’s rank correlation coefficient (ρ). A P value of < 0.050 was considered statistically significant, while P < 0.001 was considered highly significant.

## 4. Results

### 4.1. Sociodemographic and Clinical Characteristics

The mean age was 48.97 ± 8.48 years in HC, 53.87 ± 9.08 years in PD, and 51.20 ± 8.73 years in T2DM; the difference among groups was not significant (P = 0.102). Females accounted for 56.7% of the HC group, 60% of the PD group, and 66.7% of the T2DM group. Mean BMI was 27.40 ± 2.47 kg/m^2^ in HC, 28.45 ± 3.56 kg/m^2^ in PD, and 29.12 ± 5.01 kg/m^2^ in T2DM; the difference among groups was not significant. Most participants were married: 80% in the HC group, 93.3% in the PD group, and 96.7% in the T2DM group. Employment was reported by 50% of HC, 46.7% of PD, and 36.7% of T2DM participants. Urban residence predominated across all groups (86.7% in HC, 93.3% in PD, and 80% in the T2DM group) (Table 3).

**Table 3.** Sociodemographic Characteristics of the Study Participants ^a^

Sociodemographic	HC (n = 30)	PD (n = 30)	T2DM (n = 30)	P-Value
**Age (y)**				0.102
Mean ± SD	48.97 ± 8.48	53.87 ± 9.08	51.20 ± 8.73	
Minimum/maximum	35/66	36/69	34/68	
95% CI	45.8 - 52.13	50.48 - 57.26	47.94 - 54.46	
**Gender**				0.828
Female	13 (56.7)	18 (60.0)	20 (66.7)	
Male	17 (43.3)	12 (40.0)	9 (33.3)	
Male: female ratio	17:28	2:3	1:2.33	
**BMI (kg/m^2^)**				0.187
Mean ± SD	27.40 ± 2.47	28.45 ± 3.56	29.12 ± 5.01	
Minimum/maximum	23.5/34.38	22.3/36.4	20.34/46.87	
95% CI	26.48 - 28.32	27.22 - 29.88	27.24 - 30.99	
**Marital status, frequency (%)**				0.075
Married	24 (80.0)	28 (93.3)	29 (96.7)	
Single	6 (20.0)	2 (6.7)	1 (3.3)	
**Occupation**				0.557
Employed	15 (50.0)	14 (47.7)	11 (37.6)	
Unemployed	15 (50.0)	16 (53.3)	19 (63.3)	
**Residency**				0.315
Urban	26 (86.7)	28 (93.3)	24 (80.0)	
Rural	4 (13.3)	2 (6.7)	6 (20.0)	

^a^ Values are expressed as No. (%) unless otherwise indicated. Abbreviations: BMI, Body Mass Index; CI, confidence interval; HC, healthy control; PD, prediabetic; T2DM, type 2 diabetes mellitus. Age was analyzed using ANOVA, BMI using the Kruskal-Walli’s test, and categorical variables (gender, marital status, occupation, and residency) using the chi-square test.

In addition, a family history of DM was reported by 10 HC participants (33.3%), 11 PD participants (36.7%), and 12 T2DM patients (40.0%) (P = 0.866). Regarding disease duration, no value was applicable to HC, whereas PD had a mean duration of 2.27 ± 3.86 years (range, 1 month to 11 years; 95% CI, -0.33 to 4.86), and T2DM patients had a mean duration of 7.61 ± 5.86 years (range, 3 months to 20 years; 95% CI, 5.42 - 9.80), indicating a significant difference (P = 0.002). Symptoms were more frequent in the T2DM group, with 20 patients (66.7%) reporting symptoms compared with 12 individuals (40.0%) in the PD group (P = 0.038). Similarly, chronic conditions were significantly more prevalent in T2DM patients, with 21 (70%) affected versus 9 (30%) among individuals with PD (P = 0.002). Pharmacological management indicated that only 6 individuals with PD (20%) were taking oral hypoglycemics, compared with 23 T2DM patients (76.7%) (P < 0.001). Insulin therapy was exclusive to the T2DM group, with 8 patients (26.7%) using insulin (Table 4).

**Table 4.** Clinical Characteristics Among Study Groups ^a^

Clinical Characteristics	HC (n = 30)	PD (n = 30)	T2DM (n = 30)	P-Value
**Family history of diabetes**				0.866
No	20 (66.7)	19 (63.3)	18 (60.0)	
Yes	10 (33.3)	11 (36.7)	12 (40.0)	
**Duration**				0.002 ^b^
Mean ± SD (y)	-	2.27 ± 3.86	7.61 ± 5.86	
Minimum/maximum	-	1 month/11 years	3 months/20 years	
95% CI	-	-0.33 - 4.86	5.42 - 9.80	
**Symptoms**				0.038
No	-	18 (60.0)	10 (33.3)	
Yes	-	12 (40.0)	20 (66.7)	
**Chronic conditions**				0.002 ^b^
No	-	21 (70.0)	9 (30.0)	
Yes	-	9 (30.0)	21 (70.0)	
**Taking diabetes pills**				< 0.001 ^b^
No	-	24 (80.0)	7 (23.3)	
Yes	-	6 (20.0)	23 (76.7)	
**Using insulin injections**				0.002 ^b^
No	-	30 (100.0)	22 (73.3)	
Yes	-	0 (0.0)	8 (26.7)	

^a^ Values are expressed as No. (%) unless otherwise indicated. Abbreviations: CI, confidence interval; HC, healthy control; T2DM, type 2 diabetes mellitus.

^b^ Significant differences were assessed using the Mann–Whitney U test for duration and the chi-square test for categorical variables.

### 4.2. Blood Glucose Monitoring and Biochemical Parameters

**Figure 1.**
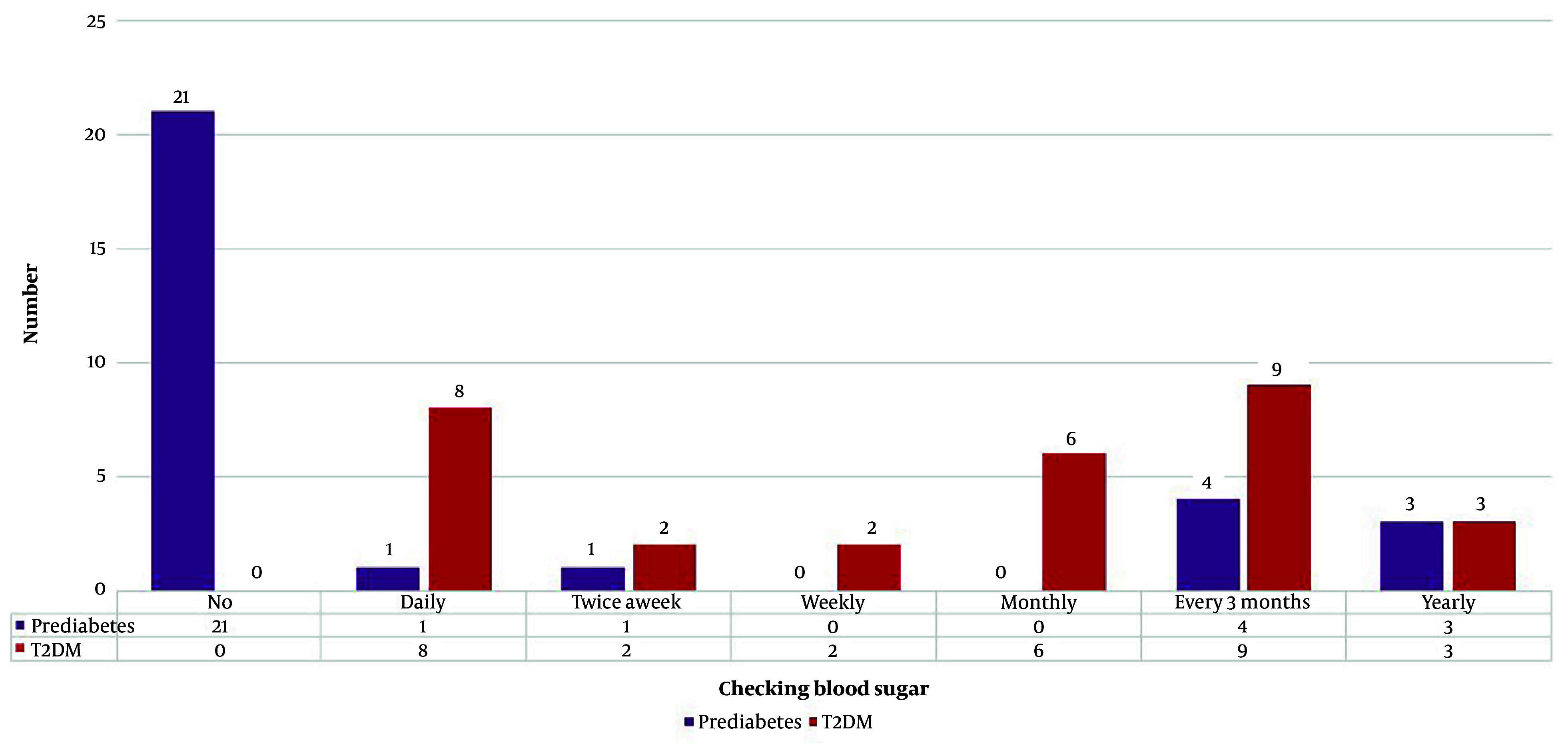
Frequency of Blood Glucose Monitoring Among Individuals with Prediabetes and Type 2 Diabetes Mellitus (T2DM). T2DM patients showed significantly more blood glucose monitoring behaviors than individuals with PD (P < 0.001).

The frequency of BG monitoring differed markedly between individuals with PD and those with T2DM. Among PD participants, most (n = 21, 70%) reported not checking their BG at all, whereas none of the T2DM patients fell into this category. In contrast, daily monitoring was reported by 8 T2DM patients (26.7%), compared with only 1 individual with PD (3.3%). Patients with T2DM demonstrated significantly more frequent and structured monitoring behaviors than those with PD (P < 0.001) (Figure 1).

Markers of glycemic control differed significantly across groups, with HbA1C levels increasing progressively from 5.39 ± 0.26% in HC to 6.11 ± 0.22% in PD and 9.21 ± 2.29% in T2DM patients; all pairwise comparisons were highly significant (P_a_ < 0.001; P_b_ < 0.001; P_c_ < 0.001). Similarly, mean RBG values were 98 ± 9 mg/dL in HC, 108 ± 25.59 mg/dL in PD, and 185 ± 101 mg/dL in T2DM (P < 0.001). C-peptide levels were highest in the PD group (3.04 ± 1.33 ng/mL), followed by HC (2.82 ± 0.72 ng/mL), and lowest in T2DM (2.60 ± 1.82 ng/mL). No significant difference was observed between HC and PD (P_a_ = 0.712); however, significant reductions were noted in T2DM compared with both HC (P_b_ = 0.012) and PD (P_c_ = 0.025). Lipid profile parameters showed no significant differences in total cholesterol (TC) (HC, 188.30 ± 27.18 mg/dL; PD, 190.52 ± 46.79 mg/dL; T2DM, 199.53 ± 43.04 mg/dL), with P_a_ = 0.842, P_b_ = 0.151, and P_c_ = 0.412, respectively. Triglyceride (TG) levels followed a similar pattern (144.30 ± 59.63, 135.05 ± 71.22, and 157.51 ± 150.04 mg/dL in HC, PD, and T2DM, respectively), with no significant differences across comparisons (P_a_ = 0.264, P_b_ = 0.228, and P_c_ = 0.340, respectively). LDL concentrations were also comparable: 124.30 ± 25.64 mg/dL in HC, 121.64 ± 42.63 mg/dL in PD, and 127.72 ± 31.24 mg/dL in T2DM, with nonsignificant differences (P_a_ = 0.771, P_b_ = 0.644, and P_c_ = 0.531, respectively). In contrast, HDL levels were significantly lower in HC (42.38 ± 10.64 mg/dL) than in PD (48.49 ± 11.51 mg/dL, P_a_ = 0.028). No significant differences were found between HC and T2DM (P_b_ = 0.061) or between PD and T2DM (P_c_ = 0.695). Urea levels were relatively consistent: 29.82 ± 8.01 mg/dL in HC, 30.61 ± 6.86 mg/dL in PD, and 27.54 ± 6.68 mg/dL in T2DM, with no significant differences across comparisons (P_a_ = 0.686, P_b_ = 0.236, and P_c_ = 0.085, respectively). Creatinine levels showed a declining trend from HC (0.82 ± 0.19 mg/dL) to PD (0.74 ± 0.15 mg/dL) and T2DM (0.65 ± 0.14 mg/dL). The difference between HC and PD was not significant (P_a_ = 0.088). Patients with T2DM had significantly lower creatinine values than both HC (P_b_ < 0.001) and PD (P_c_ = 0.021) (Table 5).

**Table 5.** Comparison of Glycemic, Lipid, and Renal Biomarkers Among Study Groups ^a, b^

Biochemical Parameters	HC (n = 30)	PD (n = 30)	T2DM (n = 30)	P_A_	P_B_	P_C_
**Glycemic control markers**						
HbA1C (mmol/mol)	5.39 ± 0.26	6.11 ± 0.22	9.21 ± 2.29	< 0.001 ^c^	< 0.001 ^c^	< 0.001 ^c^
RBG (mg/dL)	98.39 ± 9.25	108.53 ± 25.59	185.31 ± 101.29	0.019 ^c^	< 0.001 ^c^	< 0.001 ^c^
C-peptide (ng/mL)	2.82 ± 0.72	3.04 ± 1.33	2.60 ± 1.82	0.712	0.012 ^c^	0.025 ^c^
**Lipid profile (mg/dL)**						
Total cholesterol	188.30 ± 27.18	190.52 ± 46.79	199.53 ± 43.04	0.842	0.151	0.412
Triglycerides	144.30 ± 59.63	135.05 ± 71.22	157.51 ± 150.04	0.264	0.228	0.340
LDL	124.30 ± 25.64	121.64 ± 42.63	127.72 ± 31.24	0.771	0.644	0.531
HDL	42.38 ± 10.64	48.49 ± 11.51	47.10 ± 10.36	0.028 ^c^	0.061	0.695
**Renal function test (mg/dL)**						
Urea	29.82 ± 8.01	30.61 ± 6.86	27.54 ± 6.68	0.686	0.236	0.085
Creatinine	0.82 ± 0.19	0.74 ± 0.15	0.65 ± 0.14	0.088	< 0.001 ^c^	0.021 ^c^

^a^ Data are expressed as mean ± SD. Abbreviations: HbA1C, Glycated hemoglobin; HC, healthy control; HDL, high-density lipoprotein; LDL, low-density lipoprotein; PD, prediabetic; RBG, random blood glucose; T2DM, type 2 diabetes mellitus.

^b^ P values were defined as follows: P_A_ for comparisons between HC and PD, P_B_ for HC and T2DM, and P_C_ for PD and T2DM groups. HbA1C, RBG, C-peptide, total cholesterol, triglyceride, LDL, HDL, urea, and creatinine were analyzed using the Mann-Whitney U test.

^c^ Significant difference.

### 4.3. Correlation of Variables Among Patients with T2DM

A strong positive correlation was observed between HbA1C and RBG (ρ = 0.773, P < 0.001). However, C-peptide did not significantly correlate with either HbA1C or RBG. C-peptide showed a significant positive correlation with TG (ρ = 0.573, P = 0.001) and TC (ρ = 0.387, P = 0.035), suggesting that β-cell activity may be associated with lipid dysregulation in T2DM. Additionally, a strong positive correlation was found between TG and LDL levels (ρ = 0.751, P < 0.001), and a significant positive association was observed between TG and TC (ρ = 0.387, P = 0.035). In contrast, a significant inverse correlation was identified between HDL and LDL (ρ = -0.432, P = 0.017). C-peptide also showed a significant negative correlation with HDL (ρ = -0.407, P = 0.026), with no significant correlation with LDL. Creatinine levels were significantly positively correlated with both C-peptide (ρ = 0.431, P = 0.019) and HDL (ρ = 0.431, P = 0.019), whereas creatinine was not significantly correlated with other parameters. Urea did not significantly correlate with any variables (Table 6).

**Table 6.** Correlation Between Glycemic, Lipid, and Renal Markers in Type 2 Diabetes Mellitus Patients ^a^

Correlations	Glycemic Control Markers			Lipid Profile				Renal Function Test	
	HbA1C	RBS	C-peptide	TC	TG	LDL	HDL	Urea	Creatinine
**Glycemic control markers**									
HbA1C									
ρ	NA	NA							
P- Value	NA	NA							
RBS									
ρ	0.773 ^c^	NA							
P- Value	< 0.001								
C-peptide									
ρ	-0.154	-0.010							
P- Value	0.417	0.960							
Lipid profile									
TC									
ρ	0.082	0.101	-0.004						
P- Value	0.666	0.594	0.985						
TG									
ρ	-0.114	-0.061	0.573 ^c^	0.387 ^b^					
P- Value	0.547	0.751	0.001	0.035					
LDL									
ρ	-0.029	0.054	-0.125	0.751 ^c^	0.249				
P- Value	0.547	0.778	0.510	<0.001	0.185				
HDL									
ρ	-0.019	0.168	-0.407 ^b^	0.254	-0.432 ^b^	0.151			
P- Value	0.922	0.374	0.026	0.176	0.017	0.425			
Renal function test									
Urea									
ρ	0.250	0.131	-0.064	0.044	-0.015	-0.013	0.151		
P- Value	0.183	0.490	0.737	0.816	0.937	0.947	0.427		
Creatinine									
ρ	0.013	0.012	0.013	0.076	0.132	0.114	-0.196	0.431 ^b^	
P- Value	0.947	0.952	0.984	0.694	0.949	0.555	0.307	0.019	

^a^ Abbreviations: HbA1C, Glycated hemoglobin; HDL, high-density lipoprotein; LDL, low-density lipoprotein; NA, not available; RBG, random blood glucose; TC, total cholesterol; TG, triglyceride; ρ, Spearman's rank correlation coefficient. Duration was analyzed using Spearman's rank correlation test.

^b^ Correlation is significant at the 0.05 level (2-tailed).

^c^ Correlation is significant at the 0.01 level (2-tailed).

### 4.4. Molecular Analysis

**Figure 2.**
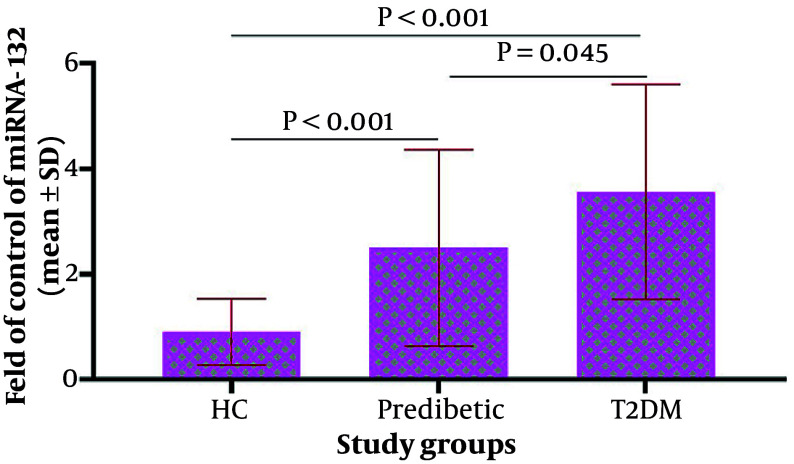
Comparative fold expression of circulating miRNA-132 across treated groups. miRNA-132 was gradually and significantly expressed (elevated) across HC, PD, and T2DM groups. HC: Healthy controls; miRNA: micro-ribonucleic acid; PD: prediabetic; T2DM: type 2 diabetes mellitus. Data were analyzed using the Mann-Whitney U test.

Quantitative analysis of circulating miRNA-132 and miRNA-126 expression levels is presented in Table 7. Expression values were normalized to U6 snRNA and calculated using the Pfaffl method (2^-ΔΔCt^), with statistical comparisons between groups. MicroRNA-132 exhibited a progressive increase across the glycemic spectrum. Healthy controls showed the lowest expression (0.90 ± 0.63; 95% CI, 0.67 - 1.14), followed by individuals with PD (2.50 ± 1.86; 95% CI, 1.74 - 3.26), with expression peaking in patients with T2DM (3.56 ± 2.04; 95% CI, 2.80 - 4.32). The differences were highly significant between HC and PD and between HC and T2DM (P < 0.001). A modest but significant elevation was also observed between PD and T2DM (P = 0.045) (Table 7 and Figure 2).

**Table 7.** Comparative Analysis of miRNA-126 and miRNA-132 Across Study Groups (N = 30) ^a^

Study Groups and Statistics	miRNA-126	miRNA-132
**HC**		
95% CI	1.03 - 1.42	0.67 - 1.14
Mean ± SD	1.22 ± 0.52	0.90 ± 0.63
**PD**		
95% CI	1.41 - 1.98	1.74 - 3.26
Mean ± SD	1.70 ± 0.74	2.50 ± 1.86
**T2DM**		
95% CI	0.97 - 1.61	2.80 - 4.32
Mean ± SD	1.29 ± 0.85	3.56 ± 2.04
**P-value**		
HC vs PD	0.021 ^b^	< 0.001 ^c^
HC vs T2DM	0.252	< 0.001 ^c^
PD vs T2DM	0.029 ^b^	0.045 ^b^

^a^ Abbreviations, CI, Confidence interval; HC, healthy control; miRNA, micro-ribonucleic acid; PD, prediabetic; T2DM, type 2 diabetes mellitus.

^b^ Significant difference.

^c^ Highly significant difference. Determined using the Mann-Whitney U test.

**Figure 3.**
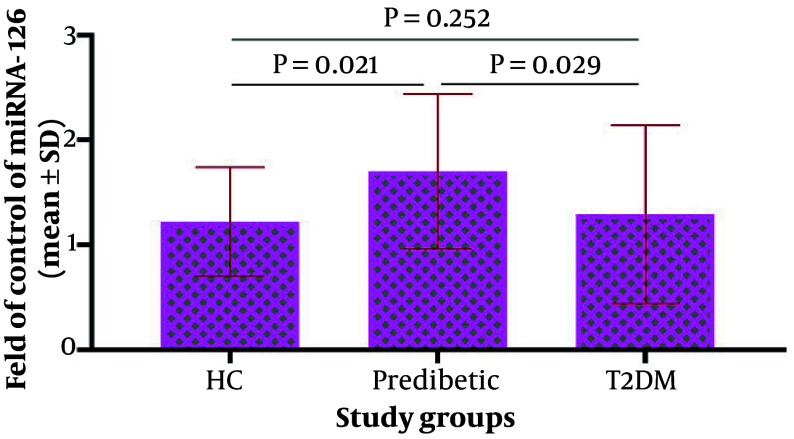
Comparative expression of circulating miRNA-126 across studied groups. A significant increase was observed in individuals with PD compared with either HC (P = 0.021) or T2DM groups (P = 0.029), while no significant differences were noted between HC and T2DM groups (P = 0.252). HC: Healthy control; miRNA: micro-ribonucleic acid; PD: prediabetic; T2DM: type 2 diabetes mellitus. Data were analyzed using the Mann-Whitney U test.

In addition, the mean expression level of miRNA-126 was lowest in the HC group (1.22 ± 0.52; 95% CI, 1.03 - 1.42), moderately elevated in T2DM patients (1.29 ± 0.85; 95% CI, 0.97 - 1.61), and highest in the PD group (1.70 ± 0.74; 95% CI, 1.41 - 1.98). A significant increase was observed in individuals with PD compared with HC (P = 0.021) and between the PD and T2DM groups (P = 0.029), while no significant differences (P = 0.252) were observed between the HC and T2DM groups (Table 7 and Figure 3).

## 5. Discussion

This study provides a comprehensive comparison of sociodemographic, clinical, and biochemical characteristics among healthy individuals, individuals with PD, and patients with T2DM. The results indicate gradual metabolic changes during the progression from normoglycemia to PD and ultimately to overt DM, with biochemical and clinical indicators proving more discriminatory than sociodemographic variables.

### 5.1. Sociodemographic and Clinical Characteristics

Sociodemographic factors were largely similar across the 3 groups. Age and sex distributions were not substantially different, suggesting that these characteristics were not primary predictors of glycemic status in this cohort. Body Mass Index showed a progressive, albeit insignificant, increase from healthy individuals to those with T2DM, indicating that although adiposity contributes to IR, the deterioration in glycemic control is influenced by other interacting factors beyond body weight alone. Marital status, occupation, and residency were comparable across groups, with most participants living in metropolitan regions. These findings suggest that sociodemographic factors did not affect glycemic categorization within this cohort, underscoring the greater diagnostic relevance of clinical and biochemical indicators. Kalra et al. also reported that gender, education, employment, socioeconomic status, BMI, and lifestyle factors are not consistently associated with DM risk, corroborating the absence of substantial sociodemographic differences observed in this study (16). Moreover, anthropometric and biochemical profiles have been reported to be comparable between urban and rural populations, further supporting the observed consistency in residency patterns across the study groups (16).

Furthermore, clinical characteristics clearly differentiated the glycemic groups. A family history of DM was prevalent across all groups, indicating its widespread occurrence in the general population. Although family history is a recognized independent risk factor for T2DM, its high prevalence suggests that it serves more as a background risk factor than as a differentiating clinical feature across glycemic categories (17). Disease duration was a significant distinguishing factor. Individuals with PD had a relatively recent onset of dysglycemia, whereas individuals with T2DM reported a much longer disease course. This trend aligns with longitudinal studies indicating that PD is an early, often transient phase, with many individuals progressing to overt DM over time (18). Symptom burden and comorbidities increased markedly from PD to T2DM. Chronic hyperglycemia in T2DM was associated with greater symptomatology and a higher prevalence of chronic comorbidities, indicating disease progression and the emergence of complications. Evidence suggests that PD substantially increases the likelihood of developing DM and related complications, whereas multimorbidity becomes more prevalent with longer T2DM duration (19, 20). Medication use corresponded with disease severity. Most individuals with PD were not receiving pharmacological treatment, whereas oral glucose-lowering medications were widely used in T2DM (21), with insulin therapy restricted to this cohort. This is consistent with clinical evidence indicating that PD is primarily managed with lifestyle modification, whereas more severe T2DM often requires pharmacological intervention because of progressive β-cell dysfunction (22).

### 5.2. Blood Glucose Monitoring and Biochemical Parameters

Distinct differences were observed in self-monitoring behavior. Most individuals with PD did not consistently check their BG levels, indicating limited engagement in preventive health measures. In contrast, individuals with T2DM reported frequent monitoring, consistent with treatment protocols and professional guidelines. These findings highlight a substantial gap in early disease management, as PD is a potentially reversible phase in which enhanced surveillance may facilitate timely intervention. Previous research indicates that individuals using insulin or combination therapy show greater adherence to glucose self-monitoring; nevertheless, overall adherence remains inadequate, corroborating the observed differences between PD and T2DM (23). In addition, RBG and HbA1C values increased significantly from healthy individuals to those with PD and T2DM, confirming worsening glycemic control across disease stages. Susairaj et al. reported similar incremental increases in glycemic indicators corresponding to elevated DM risk (24). C-peptide levels demonstrated a distinct pattern. Individuals with PD had elevated levels, presumably reflecting compensatory hyperinsulinemia due to IR (25). Conversely, individuals with T2DM had decreased C-peptide levels, indicating declining β-cell secretory capacity as the disease progresses (26). This pattern supports the conclusion that early dysglycemia is characterized by increased insulin secretion, whereas advanced T2DM is associated with progressive β-cell dysfunction (25).

Most lipid measures, including TC, LDL, and TG, showed no significant differences across groups. This may reflect variable lipid responses during early dysglycemia or the influence of cholesterol-lowering medication in individuals with T2DM. Similar findings have been reported in studies comparing normoglycemic, PD, and diabetic cohorts (27). High-density lipoprotein levels were higher in the PD cohort than in HC, whereas no significant differences were observed between PD and T2DM. Variability in high-density lipoprotein may be influenced by lifestyle factors, hormonal effects, or early metabolic adaptations rather than by glycemic status alone (27).

Urea levels were comparable across groups, indicating preserved renal function. Creatinine levels decreased gradually from healthy individuals to those with T2DM. This decrease may reflect lower muscle mass rather than enhanced renal clearance, as sarcopenia commonly occurs in chronic metabolic diseases. Findings from the Korean Sarcopenic Obesity Study support this hypothesis, reporting an increased prevalence of sarcopenia in individuals with T2DM (28).

### 5.3. Correlation Patterns Among Patients with T2DM

A strong positive correlation between RBG and HbA1C confirmed that higher daily glucose levels contribute to increased long-term glycemic burden. C-peptide showed no significant correlation with glycemic indicators, suggesting that residual β-cell activity does not directly reflect current glycemic control in established T2DM (29, 30). Triglyceride levels correlated positively with LDL and TC, consistent with diabetic dyslipidemia. C-peptide showed significant associations with TC and TG and a negative correlation with HDL, suggesting that residual insulin secretion may contribute to lipid abnormalities through IR-related pathways. The negative correlation between HDL and LDL reflects established atherogenic patterns. Aruna reported similar correlations between C-peptide levels and atherogenic lipid ratios in inadequately controlled T2DM, reinforcing the link between IR and dyslipidemia (31). Finally, creatinine showed a positive correlation with C-peptide and HDL, indicating potential associations among muscle mass, residual β-cell activity, and lipid metabolism. Urea showed no notable correlations, suggesting stable nitrogen metabolism. The strong association between T2DM and sarcopenia supports the observed creatinine-related associations (32).

### 5.4. Molecular Findings

Quantitative RT-qPCR analysis revealed upregulated circulating miRNA-126 and miRNA-132 in PD and T2DM relative to HC. MicroRNA-126 peaked in PD compared with HC and T2DM, with no HC-T2DM difference. MicroRNA-132 showed a progressive and significant increase across all groups. Liu et al. reported findings consistent with these results, observing elevated circulating miRNA-126 as a novel biomarker for screening PD and newly diagnosed T2DM (AUC, 0.78) (33). Similarly, results reported by Zeinali et al. partially aligned with our findings regarding miRNA-126 dysregulation in PD; they observed decreased circulating miRNA-126 - 3p levels compared with controls and negative correlations with inflammation in Iranian PD/T2DM cohorts. Although their pattern contrasts with ours, possibly due to cohort BMI 25 - 35 or ethnicity, the association with inflammation supports a role for miRNA-126 in the pathogenesis of early dysglycemia (34).

Zhang et al. observed significantly lower miRNA-126 in susceptible individuals with impaired fasting glucose and newly diagnosed T2DM than in normal individuals (P < 0.010) and reported an inverse association with fasting glucose. However, their signature miRNA panel confirmed the utility of miRNA-126 for early T2DM prediction, supporting miRNA-126 dysregulation during the PD/T2DM transition, as reflected by its peak expression in this study (35). Moreover, Pramanik et al. reported decreased miRNA-126 and miRNA-132 levels in plasma and vitreous humor of T2DM patients with nonproliferative diabetic retinopathy (NPDR) compared with those without diabetic retinopathy, contrasting with our observed upregulation in PD/T2DM without retinopathy (36). In contrast, Shi et al. reported decreased hippocampal miR-132 in T2DM rats via the GSK-3β/Tau pathway in a cognitive impairment model, differing from our observed plasma increase, possibly due to tissue specificity (brain vs blood), species differences (rat vs human), or hyperglycemia stage (37). Nemecz et al. reported significantly upregulated miR-132, along with miR-218 and miR-143, in microvesicles from patients with diabetic nephropathy compared with controls, linking it to vascular complications and confirming elevated circulating miR-132 in advanced T2DM, consistent with its progressive increase and metabolic/vascular role (38).

### 5.5. Limitations

This study has limitations, including its cross-sectional design, which may limit the ability to establish causal relationships among biochemical indicators, microRNA expression, and disease development across different glycemic states. The limited sample size and single-center design may also constrain the generalizability of the results to diverse populations with varying ethnic, genetic, or lifestyle characteristics. MicroRNA expression was assessed at a single time point, which may not capture longitudinal changes or temporal fluctuations during progression from PD to T2DM.

### 5.6. Conclusions

The transition from normoglycemia to PD and T2DM might be driven by metabolic and molecular changes rather than sociodemographic factors, which showed minimal discriminative value. Changes in glycemic markers and C-peptide reflect the evolving balance between IR and β-cell function, with PD characterized by compensatory hyperinsulinemia and T2DM by declining insulin secretory capacity. Correlation patterns among glycemic indices, lipid parameters, and renal markers might highlight the complex, interconnected nature of metabolic dysregulation in established DM. Circulating miRNA-126 and miRNA-132 might exhibit unique, stage-specific expression patterns across the glycemic spectrum, indicating roles in glucose metabolism, vascular regulation, and disease progression. Integrating traditional biochemical markers with molecular markers might provide a more comprehensive approach to detecting early metabolic disturbances and elucidating the continuum of DM progression.

### 5.7. Recommendations

Future research should use longitudinal designs to monitor biochemical and microRNA changes over time, thereby improving understanding of their prognostic significance for DM development. Larger, multicenter studies are recommended to confirm these results and improve their applicability to other populations. Expanding the panel of circulating miRNAs may provide a more comprehensive molecular profile associated with dysglycemia and improve biomarker sensitivity. Further investigation is needed to elucidate the molecular functions of miRNA-126 and miRNA-132 in insulin production, β-cell function, and vascular regulation, which may inform future diagnostic or therapeutic strategies.

## Data Availability

The generated data of this study are not available publicly due to its restrictive content, but can be provided by the corresponding author upon request.
